# Polymer/Iron-Based Layered Double Hydroxides as Multifunctional Wound Dressings

**DOI:** 10.3390/pharmaceutics12111130

**Published:** 2020-11-23

**Authors:** Mariana Pires Figueiredo, Ana Borrego-Sánchez, Fátima García-Villén, Dalila Miele, Silvia Rossi, Giuseppina Sandri, César Viseras, Vera Regina Leopoldo Constantino

**Affiliations:** 1 Departamento de Química Fundamental, Instituto de Química, Universidade de São Paulo—USP, Av. Prof. Lineu Prestes 748, São Paulo 05508-000, Brazil; figueiredompires@usp.br; 2 Department of Pharmacy and Pharmaceutical Technology, Faculty of Pharmacy, University of Granada—UGR, Campus of Cartuja s/n, 18071 Granada, Spain; anaborrego@iact.ugr-csic.es (A.B.-S.); fgarvillen@ugr.es (F.G.-V.); 3 Andalusian Institute of Earth Sciences, Consejo Superior de Investigaciones Científicas-University of Granada, Avenida de las Palmeras 4, Armilla, 18100 Granada, Spain; 4 Department of Drug Sciences, University of Pavia, viale Taramelli 12, 27100 Pavia, Italy; dalila.miele@unipv.it (D.M.); silvia.rossi@unipv.it (S.R.); giuseppina.sandri@unipv.it (G.S.)

**Keywords:** multifunctional dressings, skin, local therapy, layered double hydroxides, drug release modulation, wound healing, intercalation compounds

## Abstract

This work presents the development of multifunctional therapeutic membranes based on a high-performance block copolymer scaffold formed by polyether (PE) and polyamide (PA) units (known as PEBA) and layered double hydroxide (LDH) biomaterials, with the aim to study their uses as wound dressings. Two LDH layer compositions were employed containing Mg^2+^ or Zn^2+^, Fe^3+^ and Al^3+^ cations, intercalated with chloride anions, abbreviated as Mg-Cl or Zn-Cl, or intercalated with naproxenate (NAP) anions, abbreviated as Mg-NAP or Zn-NAP. Membranes were structurally and physically characterized, and the in vitro drug release kinetics and cytotoxicity assessed. PEBA-loading NaNAP salt particles were also prepared for comparison. Intercalated NAP anions improved LDH–polymer interaction, resulting in membranes with greater mechanical performance compared to the polymer only or to the membranes containing the Cl-LDHs. Drug release (in saline solution) was sustained for at least 8 h for all samples and release kinetics could be modulated: a slower, an intermediate and a faster NAP release were observed from membranes containing Zn-NAP, NaNAP and Mg-NAP particles, respectively. In general, cell viability was higher in the presence of Mg-LDH and the membranes presented improved performance in comparison with the powdered samples. PEBA containing Mg-NAP sample stood out among all membranes in all the evaluated aspects, thus being considered a great candidate for application as multifunctional therapeutic dressings.

## 1. Introduction

Multifunctional therapeutic dressings that are able to act in the tissue regeneration process, besides play-acting as physical protective barriers against infection, occupy a high level of interest in pharmaceutical technology. Multifunction is often associated with multicomponent which may originate systems that are too complex, making understanding and controlling the device’s properties difficult. Thus, dressings containing materials such as layered double hydroxides (LDHs), multifunctional on their own, may contribute to the design of simple and effective devices. LDHs possess general formula [M^2+^_(1−X)_M^3+^_X_(OH)_2_](A^n−^)·zH_2_O, being composed by a mixture of di (M^2+^) and trivalent (M^3+^) metallic cations that, in controlled conditions, precipitate as stacked, positively charged layers. Structural neutrality of LDH is guaranteed by the presence of hydrated anions (A^n−^) between the layers [1,2]. Among many uses, such as catalysts [3,4], magnetic materials [5] and polymer fillers [6], LDHs stand out for their application as vehicles for drug storage and delivery [7,8], mainly due to their in vitro and in vivo biocompatibility [9,10,11,12,13,14] and to the possibility to modify drug release kinetics. Anti-inflammatories [15,16] and bactericidal [17,18,19] species are examples of organic guests, already loaded into LDH, that can promote a faster and painless wound healing process. Briefly, such process is characterized by a sequence of four complex, overlapped and regulated phases: hemostasis, inflammation, proliferation and remodeling [20]. The LDH skeleton role in the tissue regeneration process has started being studied. LDHs may experience a process of M^2+^ and M^3+^ cations leaching, especially in acid media such as the skin (pH value range 4–7 [21]). Some of us have reported the role of LDHs on in vivo collagen neogenesis modulation according to different M^2+^ and M^3+^ cations (and chloride as intercalated anions), verified through intramuscular implantation of LDH tablets in rats [10,11]. For instance, type-III collagen is mainly formed in the presence of Zn^2+^ and Al^3+^, while type-I predominates by changing Zn^2+^ by Mg^2+^ cations. By replacing half of the Al^3+^ content by Fe^3+^, type-I collagen prevailed for both LDHs, either composed by Mg or Zn. Therefore, LDH scaffold possesses a biological role in the tissue regeneration process and the composition of the materials may be chosen according to the interest in the type of collagen to be formed in specific locals of application, tissue characteristics and treatment. According to the literature, LDHs studied as drug carriers are mostly formed by Mg^2+^ and Zn^2+^ as divalent cations and by Al^3+^ as exogenous ion [12]. Differently from endogenous metals, aluminum elimination is not regulated and its accumulation in different tissues has been verified [22,23]. The partial or complete substitution of Al^3+^ by Fe^3+^ on LDH compositions may originate more suitable compositions for their application as biomaterials. 

**Figure 1:**
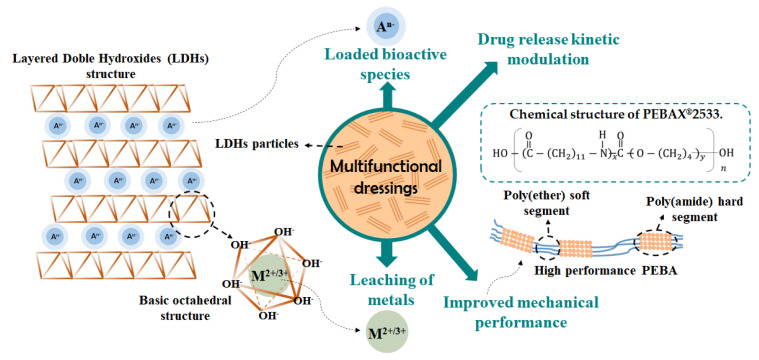
Schematic representation of the designed multifunctional dressings, its multiple components, and the schematic representation of the general structure of layered double hydroxide (LDH) and polyether (PE) and polyamide (PA), also known as PEBA, polymer.

Modern dressings are mostly composed by natural polymers, such as carboxymethylcellulose, gelatin and pectin, or synthetic polymers, including polyurethane, silicon, poly(methacrylates), polyvinyl pyrrolidine and nylon [24]. Mechanical resistance is an important feature to consider when choosing a polymer to compose solid dressings. PEBA comprises a family of synthetic thermoplastic elastomer block copolymers formed by polyether (PE) and polyamide (PA) units, as illustrated in Figure 1. PEBA has attracted attention due to its interesting mechanical properties and is able to be easily modulated since it can be formed by a large number of chemical structures [25]. While PA blocks are hard, PE portions act as a soft phase [25]. PEBAX^®^MED products were developed for medical applications such as anti-static additives, breathable membranes, transdermal patches, catheters and angioplasty balloons [26,27]. Some characteristics that make PEBAX^®^MED promising to medical applications include the facility to be sterilized, resistance to chemicals, high selectivity, permeability, and long-term stability [28].

The membranes developed in this work to be applied as multifunctional therapeutic dressings aim to achieve a faster and painless wound healing process. Designed dressings were based on PEBAX^®^2533 (Figure 1), composed of 80 wt% of poly(tetramethylene oxide) and 20 wt% of poly(amide)-12, and iron-based LDHs intercalated with naproxenate anions (NAP), a model drug anion derived from the non-steroidal anti-inflammatory (NSAID). 

Dressings containing NAP may relieve mild to moderate pain during wound closure due to its analgesic effect. Topical administration of NSAID is interesting to overcome the gastrointestinal side effect arising from oral administration route [29]. The most severe cases include abdominal pain, nausea and gastric ulcers [30]. Local administration may increase local concentration of the drug in subjacent tissues, such as muscles, not being discarded its systemic action once reaching the bloodstream [31]. Transdermal release of naproxen and its sodic form has been studied in gel-like formulation [32], polymeric films [33,34], polymeric nanoparticles [35] and microemulsion [36]. Besides the possibility to modulate NAP release and to provide M^2+^/M^3+^ cations to assist wound closure, LDHs particles may also improve even more the mechanical performance of PEBA polymeric support. Figure 1 shows the schematic representation of the LDH structure and its role in the multifunctional developed membranes. Once LDHs, composed by both Mg^2+^ and Zn^2+^ cations, have shown good results for biological applications, two-layer compositions, Mg_4_FeAl and Zn_4_FeAl, were applied. Dressings containing NaNAP or LDHs intercalated with NAP were tested. Furthermore, pristine PEBA and membranes containing pristine LDHs (intercalated with Cl^−^ anions) were also studied to evaluate the effect of NAP on properties of LDH–PEBA composites. Potential dressings were structurally characterized, and their mechanical resistance, in vitro drug release profile, and cytotoxicity were assessed. To the best of our knowledge, this work presents for the first time the design of composites based on PEBAX^®^ and LDH particles as well as the development of PEBAX^®^ dressings able to perform a modified drug release. 

## 2. Materials and Methods

### 2.1. Experimental Material

PEBAX^®^2533 (PEBA) containing 80 wt% of poly(tetramethylene oxide) and 20 wt% of poly(amide 12) portions was obtained from Arkema S.A. (Paris, France). Magnesium chloride hexahydrate (MgCl_2_·6H_2_O) (99%), aluminum chloride hexahydrate (AlCl_3_·6H_2_O) (99%), iron(III) chloride hexahydrate (FeCl_3_·6H_2_O) (98%), sodium hydroxide (NaOH) (≥98%) and sodium naproxenate (NaNAP) (NaC_14_H_13_O_3_) (≥98%) were purchased from Sigma-Aldrich (St. Louis, MO, USA). Thiazolyl blue tetrazolium bromide (MTT) (98%), sodium chloride (NaCl) (99%) and fetal bovine serum (PBS) (cod. F2442) were purchased from Sigma-Aldrich (Milan, Italy). 2-propanol PA was purchased from Synth (Diadema, Brazil). DMEM High Glucose (4500 mg L^−1^ glucose, l-glutamine, sodium bicarbonate and without sodium pyruvate) was purchased from Microtech^®^ (Milan, Italy). Solution containing 1% amphotericin/streptomycin/penicillin solution was purchased from EuroClone^®^ (Milan, Italy). Normal human dermal fibroblast (NHDF) juvenile foreskin (cod. C-12300) were purchased from Promocell^®^ (Heidelberg, Germany). All products were used as received. 

### 2.2. Synthesis of Mg_4_FeAl and Zn_4_FeAl LDHs Intercalated with Cl^−^ or NAP Anions

Materials with layer compositions Mg_4_FeAl and Zn_4_FeAl were abbreviated according to the divalent cations and the intercalated anion, Cl^−^ or NAP, as follows: Mg-Cl, Mg-NAP, Zn-Cl or Zn-NAP (specifically) and Cl-LDH or NAP-LDH (generally). Mg-Cl and Mg-NAP samples were synthesized by the same methods previously reported [37]. Zn-Cl and Zn-NAP LDHs were prepared by the same method with the exception of the temperature value (kept at room temperature for both pristine and hybrid phases) and the NAP/(Fe^3+^ + Al^3+^) molar ratio kept equal to 1 for intercalation of NAP by ion-exchange reaction.

### 2.3. Preparation of Pristine PEBA and PEBA Composite Membranes

Composites were designed to have an LDH percentage as high as possible whilst also maintaining visual homogeneity and resistance to handling. As shown in Table 1, membranes containing NAP, as sodium salt or loaded into LDH, were prepared, as briefly reported previously [38], aiming to have approximately the same drug amount (about 4 wt%). For each layer composition, the same weight percentage for pristine and hybrid LDH was applied, to favor comparison. 

#### 2.3.1. Preparation of Pristine PEBA Membrane

PEBA membrane was prepared by casting method, dissolving polymer beads in 2-propanol in a 3 wt% concentration under stirring at 80 °C for 2 h. Polymer solution was transferred to a Teflon sample holder with cylindrical internal cavity with 2 cm of diameter and 0.7 cm of height. Solvent evaporation was performed in a fume hood for at least 8 h.

#### 2.3.2. Preparation of PEBA Membranes Containing NaNAP Species

First of all, it was performed with the polymer dissolution, as described above. Then, the amount of NaNAP necessary to obtain the NAP percentage mentioned in Table 1 was dispersed in the polymer solution under stirring. Solvent evaporation was carried out as applied for the PEBA membrane. Sample was abbreviated as PEBA_NaNAP.

#### 2.3.3. Preparation of PEBA Membranes Containing LDH Particles

**Figure 2:**
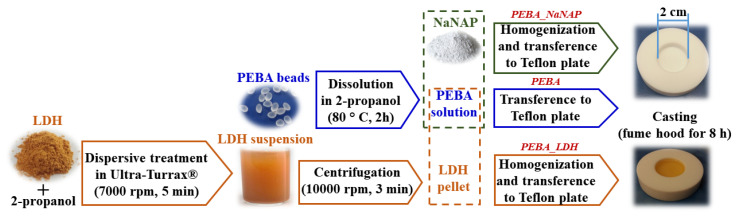
Schematic representation of PEBA membrane and composite preparation processes.

LDHs were previously treated to improve suspension stability and homogeneity of particles distribution. The respective amounts of LDH to reach the same drug percentage encapsulated in the composites, shown in Table 1, were suspended in 5 mL of 2-propanol. Suspensions were submitted to Ultra-Turrax IKA^®^ T18 (Staufen, Germany) at 7000 rpm for 5 min. Solids were isolated through centrifugation at 10,000 rpm for 3 min, resuspended in the 3 wt% PEBA solution and manually homogenized. Suspensions were transferred to Teflon plates and the solvent evaporation was carried out as described previously. Samples were abbreviated as PEBA_LDH or, more specifically, according with the composition of the LDH, as follows: PEBA_Mg-Cl, PEBA_Mg-NAP, PEBA_Zn-Cl and PEBA_Zn-NAP. Figure 2 presents a schematic representation of PEBA membrane preparation.

#### 2.3.4. In Vitro NAP Release Assays

Naproxen release experiments were conducted in quintuplicates using 2 cm diameter circular membranes. Membranes were directly accommodated in Franz’s cells with no additional membrane since it is intended to mimic wounds. Franz receptor chambers were filled with 6.4 mL of saline solution (0.9 wt% NaCl), pH value equal to 7.22 ± 0.01, simulating human plasma. The release media was constantly homogenized with stir-bar. At predefined times, 0.8 mL of the media was collected and immediately replenished with fresh saline medium. NAP concentration was determined by UV−visible absorption spectrophotometry at a maximum absorption (λ_max_) equal to 230 nm. Quantification was made by means of a calibration curve (*R*^2^ = 0.999) build with minimal and maximal NaNAP solutions with concentrations equal to 0.971 and 2.200 ppm, respectively. After the first released NAP aliquot removal, accumulative NAP weight released at each time was corrected, taking into account the NAP amount present in each subsequent aliquot removed for the measurements. Metals were quantified at the end of the assay by inductively coupled plasma atomic emission spectroscopy (ICP-AES).

#### 2.3.5. Cells Viability Evaluation: MTT Assay

Cytotoxicity induced by the membranes was assessed by an MTT test in 96-well plates (Cellstar 96 Well Culture Plate, Greiner Bio-One, Kremsmünster, Austria). Membranes were cut as circles with the correspondent well’s area (0.32 cm^2^). For comparison, an equivalent amount of LDH and NaNAP samples in powder present in the composites (Table 1) were also evaluated, as well as pristine PEBA membrane. NHDFs were seeded onto membranes in well plates (0.35 × 10^5^ cell/well in 200 µL/well) and incubated for 24 h at 37 °C in humidified atmosphere containing 5% CO_2_ (CO_2_ Incubator, PBI International, Milano, Italy). Powdered samples were added after cell confluence and then the same MTT procedure was applied for all the samples. Briefly, medium was removed after 24 h of treatment, cells were washed with PBS (10% *v*/*v*, 200 µL/well), exposed to 50 µL MTT solution (2.5 mg ml^−1^ solubilized in DMEM w/o red phenol) diluted in 100 µL of DMEM (w/o red Phenol) and incubated for 3 h at 37 °C. Next, the MTT reagent was removed and 100 µL of DMSO was added into each well to lyse cells. Finally, the absorbance was read at 570 nm with 690 nm as wavelength reference by means of an ELISA plate reader (Imark Absorbance Reader, Biorad, Milan, Italy). Cell viability was calculated as the percentage ratio between the absorbance of each sample and the absorbance of controls (cell substrates in growth medium). 

### 2.4. Material Characterization

The carbon percentage was determined on a Perkin Elmer-CHN 2400 equipment at the Central Analítica of Instituto de Química of the Universidade de São Paulo (USP).

Inductively coupled plasma atomic emission spectroscopy (ICP-AES) analysis was applied for metal quantification in synthesized LDHs, in a Spectro Analytical Instrument (Kleve, Germany) at the Central Analítica of Instituto de Química of USP. The quantification of metal cations from the release media after in vitro drug release assay was performed in a Perkin-Elmer optima 8300 instrument (PerkinElmer, Waltham, MA, USA) at the Centro de Instrumentación Científica of the University of Granada (UGR).

X-ray diffraction (XRD) patterns were obtained on a Rigaku Ultima Plus equipment (Tokyo, Japan), with Bragg–Brentano geometry and graphite crystal monochromator, using Cu-Kα radiation (1.5406 Å), 30 kV, 15 mA, Ni filter, scan range 1.5–70°(2θ) and scan step of 0.05°(2θ)/2 s.

Average particle size determination was conducted in 2-propanol through low angle laser light scattering in a Mastersize 200 Malvern equipment (Worcestershire, United Kingdom) at the Laboratório de Caracterização Tecnológica (LCT) of the Escola Politécnica of USP.

Differential scanning calorimetry (DSC) was carried out at the Central Analítica of Instituto de Química of USP using a TA instruments—Q10 equipment (New Castle, DE, USA) under 50 mL min^−1^ N_2_ flow, a heating/cooling rate of (±) 10 °C/min, using alumina hermetic and close crucible. Samples were first equilibrated at −70 °C and then submitted to a heating cycle until 100 °C (in order to erase information coming from the preparation procedure), a cooling cycle until −70 °C and a second heating cycle until 100 °C. 

Mass spectrometry coupled to thermogravimetric analyses (TGA–MS) were recorded on a Netzsch thermoanalyzer model TGA/DSC 490 PC Luxx (Spectro Analytical Instruments GmbH, Selb, German) coupled to an Aëolos 403 C mass spectrometer, using alumina crucible and heating rate of 10 °C min^−1^ under synthetic air flow of 50 mL min^−1^. 

Fourier transform infrared (FT-IR) spectra were recorded in the 4000−400 cm^−1^ range on a Bruker spectrophotometer (Billerica, MA, USA), model alpha by ATR with acquisition step of 4 cm^−1^ and 512 scans. 

Images by optic microscopy were registered in an optic microscope Coleman DN-107T (Santo André, Brazil).

Images from scanning electron microscopy (SEM) were obtained at the Central Analítica of Instituto de Química of USP in an FE-SEM Jeol JSM 7401F (FREG) equipment (JEOL, Tokyo, Japan) applying membranes deposited on a copper tape covered with gold.

Dynamo-mechanical analysis (DMA) was performed at the Central Analítica of Instituto de Química of USP on a DMA Q800 TA instrument (New Castle, DE, USA). Each PEBA membrane was cut into 5 rectangles with 3 × 0.5 cm and the analysis was performed at 70 °C. 

Membrane thicknesses were determined using a Mitutoyo analogical micrometer. Five measurements in different points were performed in 2 cm diameter circular membranes. 

Static water contact angle measurements were performed in triplicate with static deionized water drop on the membranes using SEO portable equipment (Surface Electro Optics Co., Ltd., Phoenix-I, Korea).

Absorbance in the ultraviolet–visible region was measured on a Perkin Elmer Lambda 25 spectrophotometer (Perkin Elmer, Waltham, MA, USA).

NAP release experiments were conducted on a BioScientific Inc Franz diffusion cells system (FDC40020FF, Phoenix, AZ, USA) with contact area equal to 0.63 cm^2^.

## 3. Results

### 3.1. Sample Characterization

Preliminary results of SEM, XRD and vertical mechanical resistance of some membranes were first partially published on the annals of the 15° CBPol Congress [38]. In this work, results concerning the membrane characterization were revisited, enhanced and complemented. 

Table S1 presents the chemical composition of the LDHs. Metal molar ratios are close to the expected values according with the salt’s concentration in the mother solution. An appreciable amount of carbon is verified for both Mg_4_FeAl-NAP and Zn_4_FeAl-NAP LDHs, whose NAP percentage corresponds to 36.35% and 26.64% of the material’s mass, respectively. 

**Figure 3:**
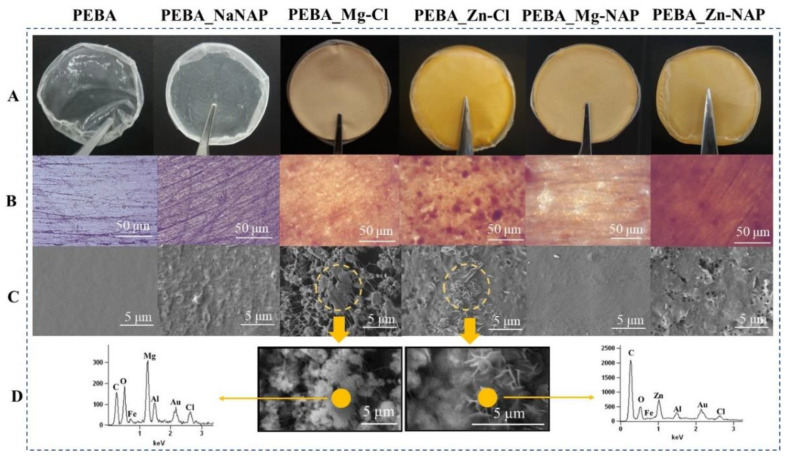
Samples are disposed in the columns. Line (**A**) macroscopic aspects, line (**B**) optical microscopy images, (**C**) SEM micrographs and (**D**) EDS spectra.

Figure 3 presents surface aspects of the polymeric samples in different scales. Samples are disposed in the columns. In line A, showing macroscopic images, it is possible to verify that membranes are visually homogeneous. Pristine PEBA is translucent while PEBA_NaNAP is cloudy. Membranes turn opaque and acquire an orange color when LDH particles are present. In line B (optical microscopy images), Teflon-plate surfaces is not perfectly smooth, originating the wrinkles clearly visualized for PEBA and PEBA_NaNAP samples. For PEBA_Mg-Cl, PEBA_Mg-NAP and PEBA_Zn-NAP membranes, particles are more homogeneously dispersed into the polymer compared to the PEBA_Zn-Cl sample, in which it is possible to clearly see darker points of particles aggregation. Line C (SEM micrographs) shows a smooth surface for pristine PEBA and a rough surface for the PEBA_NaNAP sample, due to NaNAP particle aggregation. For the PEBA_Mg-Cl sample, LDH particles are surrounded by polymer fibers. Instead, for the PEBA_Zn-Cl sample, the plate-like association of LDH particles is visualized and is similar to the morphology observed for powder LDHs [11,37]. For the PEBA_Zn-NAP sample, surface fill failures are observed and LDH particle aggregation is no longer visualized. The PEBA_Mg-NAP sample is compared to that of pristine PEBA. For the peaks in EDS spectra (line D), the indicated yellow points match the composition of the LDH layers (Mg^2+^ or Zn^2+^, Fe^3+^ and Al^3+^ cations), the intercalated anion (Cl^−^) and the composition of the polymer (C and O). Characteristic emission of Au refers to samples recovered.

Table 2 presents the average thicknesses of the membranes. Composite membranes are thicker than pristine PEBA (82 ± 14 µm). The PEBA_NaNAP sample experienced a light increase in thickness (90 ± 10 µm). Compared to the PEBA_Mg-Cl (104 ± 1 µm) and PEBA_Zn-Cl (103 ± 5 µm) samples, the presence of LDH particles intercalated with NAP resulted in thinner membranes (thicknesses equal to 86 ± 6 and 96 ± 6 µm for the PEBA_Mg-NAP and PEBA_Zn-NAP samples, respectively). 

Static water contact angle (SWCA) values for pristine PEBA and composites (see also Figure S1) are shown in Table 2. SWCA for pristine PEBA is very close to the value reported for a PEBAX^®^2533 membrane also prepared by casting method (81 ± 2° [39]). PEBA_LDH membranes presented lower SWCA values indicating an increase in surfaces hydrophilicity. PEBA_NaNAP membranes presented the lowest SWCA value (60 ± 2°), an expected behavior due to the presence of particles of an organic salt relatively soluble in water (0.5504 ± 0.0012 mol dm^−3^ at 293.15 K [40]).

Mass percentage of LDHs in the membranes are comparable, 10 wt% for LDHs composed by Mg^2+^ and 13 wt% for LDHs composed by Zn^2+^ cations. In addition, Figure S2 shows that the apparent volumes occupied by the same mass of LDH are visually similar. In order to better understand the effect of macroscopic characteristics related to the different material compositions in the homogeneity of the composite membranes, LDH particles size distributions were evaluated (Figure S3) in the same solvent applied in previous particle dispersive treatment and for composite preparation. The Zn-Cl LDH average particles size is higher than that of the Mg-Cl LDH. Pristine LDHs present higher average particle sizes compared to LDHs intercalated with NAP. Although 50% of the particles have a size up to 6.928 ± 0.001 µm for the Zn-NAP, against 9.631 ± 0.001 µm for the Mg-NAP sample, the polydispersity of the Zn-NAP sample compared to the other LDH compositions is higher, where the presence of two curves of the particle distribution with well-defined maximums is clear. 

**Figure 4:**
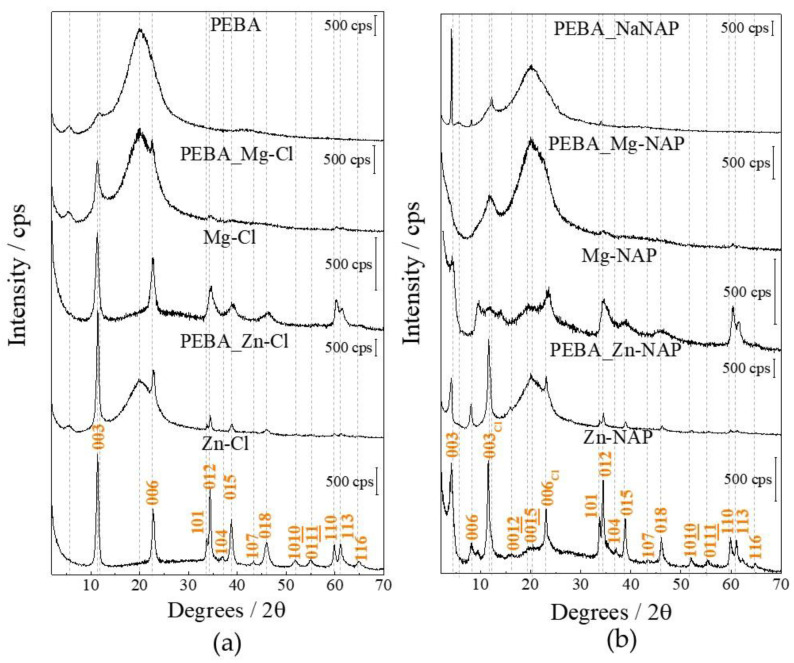
XRD patterns of pristine PEBA and PEBA composites containing Cl-LDHs (**a**) and composites containing the NaNAP salt and NAP-LDHs (**b**).

Figure 4a shows the XRD patterns of pristine PEBA and PEBA composites plus pristine LDHs, while Figure 4b exhibits the profiles of composites containing the NaNAP salt and hybrid organic–inorganic LDHs. In Figure 4a, pristine PEBA possesses a semi-crystalline structure presenting a highly intense and broad peak at 20°(2θ), similar to the literature [41] and to the XRD pattern of poly(amide 12) [42], corresponding to the more abundant block portion in PEBA. The indexation of diffraction peaks of LDHs was made considering the 3R1 polytype, mostly observed for LDHs. Basal spacing related to the (003) plane for both Mg-Cl and Zn-Cl materials is equal to 7.8 Å. As usually observed, Zn^2+^ cations promoted a higher structural organization of the LDH structure than the magnesium ions [11,43]. The most intense LDH peaks are present in the XRD pattern of the PEBA_LDH composites. According to Figure 4b, the PEBA_NaNAP sample presents intense and narrow peaks assigned to the sodic salt. Compared to the Cl-LDHs, NAP intercalation shifts the (00l) reflections to smaller angle (2θ) region, indicating larger interlayer distances. Basal spacing related the (003) plane is equal to 21.0 and 21.8 Å for the Mg-NAP and Zn-NAP LDHs, respectively. For both hybrid LDHs, the (003) and (006) harmonic peaks attributed to the Cl-LDH phases are also present. The permanence of the (113) peak, dependent on the c axis and, therefore, expected to be dislocated to small angle region with the intercalation of a much more bulk anion, is another indication of the presence of a mixture of phases. These characteristics have also been observed for NAP loaded into LDH with different layer compositions and prepared by other methods besides ion-exchange reaction [11,41,42] (such as coprecipitation [44] or reconstruction [15]), therefore being considered a peculiarity of the NAP-LDH system. Differently from the PEBA_Zn-NAP membrane, whose reflections associated to the Zn-NAP LDH are clearly observed, XRD pattern of the PEBA_Mg-NAP sample shows only the broad peaks of the organic polymer. 

Stress at break, strain at break and Young modulus of membranes submitted to tensile tests are shown in Table 2. With the exception of the Zn-Cl LDH, all particles improved the stress at break of the composites. The PEBA_Mg-NAP sample (829 ± 45 kPa) can be highlighted for having approximately twice the average tension at break in relation to pristine PEBA (479 ± 27 kPa). PEBA_Mg-NAP membrane also presented, as well, the higher elongation value (55 ± 16%) among all samples. Pristine PEBA membrane and membranes containing Mg-Cl or Zn-Cl LDH particles presented a similar rigidity, with Young modulus values equal to 3.5 ± 0.9, 3.9 ± 0.5 and 3.4 ± 0.4 MPa, respectively. On the other hand, NaNAP particles conferred a considerable stiffening to the membrane, with Young modulus equal to 7.4 ± 0.8 MPa. PEBA_Mg-NAP and PEBA_Zn-NAP samples presented an intermediate stiffness, with Young modulus values equal to 5.3 ± 0.7 and 5.2 ± 0.7 MPa, respectively.

FT-IR spectra of pristine PEBA and PEBA composites containing Cl-LDHs and the composites containing the NaNAP salt and NAP-LDHs are shown in Figure S4a,b, respectively; the bands tentative assignment is compiled in Table 3. For PEBA (wavenumbers indicated in blue in Figure S4a), the PA portion originates the bands at 3300, 1638, 1541 and 1242 cm^−1^, attributed to hydrogen bounded amide group, and also the band at 720 cm^−1^ assigned to the CH_2_ bending of the alkyl portion [28,39,41,45,46,47,48]. The strong band at 1104 cm^−1^ is assigned to the C–O–C stretching of ether group (PE portion). The bands at 2920, 2850, 1467 and 1447 cm^−1^ are attributed, respectively, to the antisymmetric stretching, symmetric stretching, bending and scissoring of the CH_2_ group of both hard and soft segments. The band at 1368 cm^−1^ is assigned to the bending of the C–C–H group while the band at 1735 cm^−1^ is related to the ester linking group. The bands at 3400, 1360 and bellow 700 cm^−1^ (orange wavenumbers indicated in the spectra of Zn-Cl and Zn-NAP samples) are attributed to the O–H stretching of the hydroxyl groups in the LDH layers and of superficially adsorbed and intercalated water molecules, to the antisymmetric stretching of CO_3_^−2^ (ν_3_) and to M–OH translations, respectively [1]. The band at 1624 cm^−1^ is assigned to angular deformation of water molecules [49]. The spectrum of the PEBA_NaNAP sample is very similar to that of pristine PEBA. The bands at 1393, 1162 and 925 cm^−1^ are related to the vibrational modes of NAP (shown in green) [11] and attributed to the stretching of the C–C=C group: C–C stretching in the naphthalene ring and CH_3_ wagging, C–O and C–H stretching, respectively. Additionally, both bands at 925 and 815 cm^−1^ can be assigned to the =C–H bending out of plane [49].

**Figure 5:**
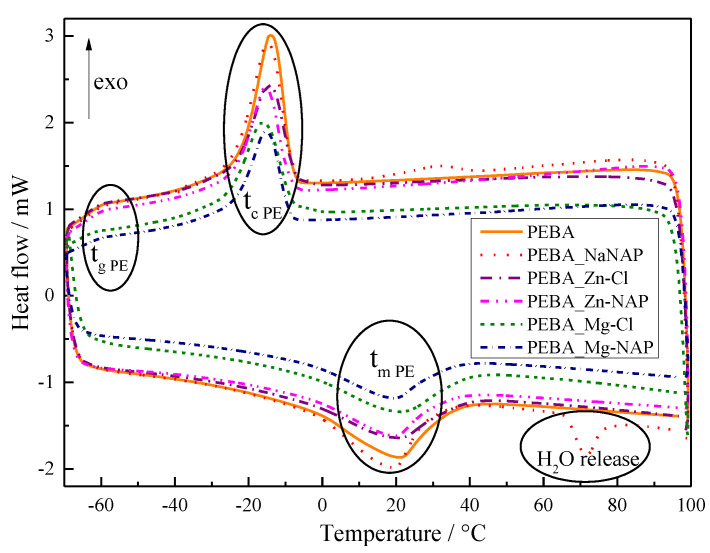
Cooling (top) and second heating (bottom) differential scanning calorimetry (DSC) scans for pristine PEBA and PEBA composites. T_g PE_—glass transition temperature of PE portion, T_c PE_—crystallization temperature of PE portion, T_m PE_—melting temperature of PE portion.

Figure 5 shows the cooling (top) and second heating (bottom) DSC scans for pristine PEBA and PEBA composites, whose peak temperature values related to the thermal events are gathered in Supplementary Materials Table S2. The temperature of glass transition for the PE portion (Tg PE) varied for all samples between −59 and −60 °C, as reported [50]. Crystallization peak temperature values of the PE portion ranged from −14 to −16 °C, also consistent with the literature [50], and slightly decreased with the presence of particles. Peak melting temperature value for PE (T_m_ PE) is normally observed around 0 and 20 °C [51]. No considerable variation in T_m_ PE was verified among the samples, ranging from 19 to 21 °C. Upper temperature for DSC analysis was carefully limited to ensure that no particle degradation would take place. Therefore, it was only possible to obtain information about thermal events occurring in the PE portion (portion present in much greater quantity in PEBAX^®^2533), since the peak melting temperature (T_m_) of PA is expected at around 140 °C or above [51]. Additionally, the endothermic event appearing around 72 °C for the PEBA_NaNAP sample is related to the release of water from salt particles [52].

Considering the application of dressings on wound and the importance of material toxicity, residual solvent fixation on the PEBA chain after pristine membrane preparation was investigated by TGA–DTG–MS analyses (Figure S5). If present, the release of residual solvent is expected in the low temperature region, around 2-propanol boiling point (82 °C [53]). However, until approximately 200 °C, no weight loss occurs. From 200 °C, a release of a fragment with *m/z* ratio equal to 60 is observed. To confirm if the signal *m/z* = 60 is associated with the 2-propanol release, possibly delayed due to the interaction with the polymer chains (and/or to a decomposition fragment), pristine PEBA pellets were also analyzed (Figure S6). In this case, a signal *m/z* = 60 was in fact observed, the peak slightly dislocated to low temperature values. The different shapes of the samples could plausibly explain the temperature shift due to differential heat transfer.

The design of multifunctional dressings requires the understanding of the structure and properties of the final materials, as well as of each component separately. It was shown above the structural and compositional characterization of the applied LDHs, and the structural, compositional, textural and thermal characterization of pristine PEBA and PEBA composite membranes. Next, the in vitro drug performance and in vitro biological response of the multifunctional dressings will be shown. 

### 3.2. In Vitro NAP Release Profile and Kinetics

**Figure 6:**
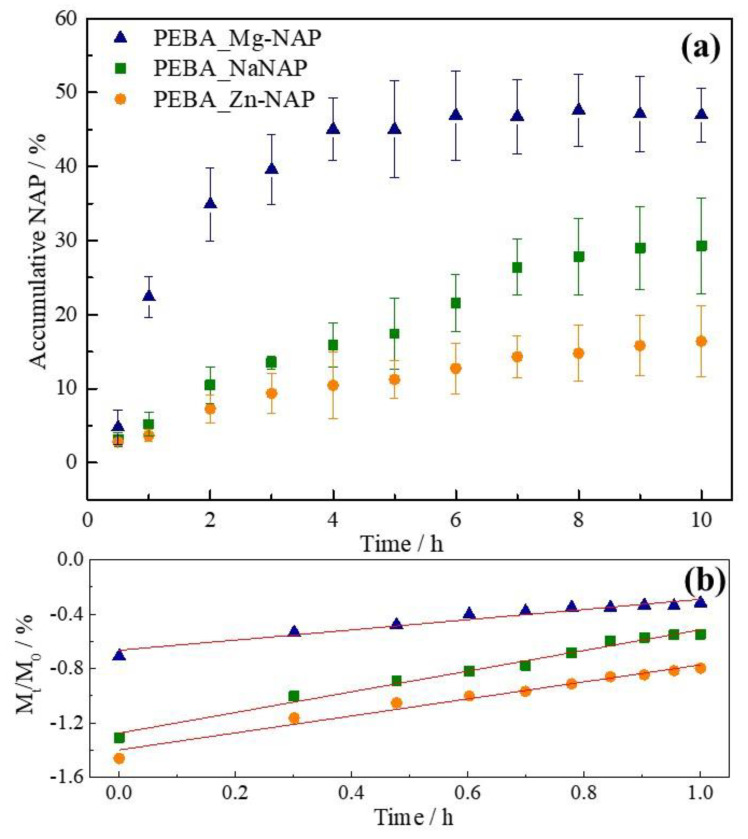
(**a**) Release profile of PEBA_NaNAP, PEBA_Mg-NAP and PEBA_Zn-NAP samples and (**b**) log–log scale for application of Power Law model.

Figure 6a presents the profiles of NAP released from the PEBA_Mg-NAP, PEBA_Zn-NAP and PEBA_NaNAP samples in saline solution. After 10 h, 47 and 16% of NAP were released from PEBA_Mg-NAP and PEBA_Zn-NAP samples, respectively. The PEBA_NaNAP sample shows an intermediate behavior in which 29% of NAP was released after 10 h. The release of leached metals after the end of the tests (10 h) was observed: the amount of Mg and Zn were 2.2 and 0.9 mg L^−1^, corresponding to 12.2% and 2.0% of the metal content in the membranes, respectively. The number of trivalent cations was below the limit of detection. 

Among the most applied kinetic drug release models (e.g., zero-order, first-order, Hixson–Crowell), the Power Law Ritger–Peppas model (also called Power Law) is considered the most appropriate to evaluate drug release from polymeric systems [52]. Power Law is described by Equation (1), where M_t_ equal the released drug at a certain time (t) and M_0_ the total loaded drug. Figure 6b shows the plot of Power Law kinetic model that resulted in a satisfactory linear correlation (*R*^2^ values close to 1) for all samples, as can be seen in Table 4. The lower n value obtained for the PEBA_Mg-NAP sample indicates a Fickian type release in which drug release is governed by diffusion, even more notably than polymeric chain relaxation. For the PEBA_Zn-NAP and PEBA_NaNAP samples, 0.5 < n < 1.0 and the drug release is characterized by a non-Fickian or anomalous transport, and both solvent diffusion and polymeric chain relaxations are relevant events.

M_t_/M_0_ = kt^n^, (1)

### 3.3. Cell Viability Evaluation: MTT Assay

**Figure 7:**
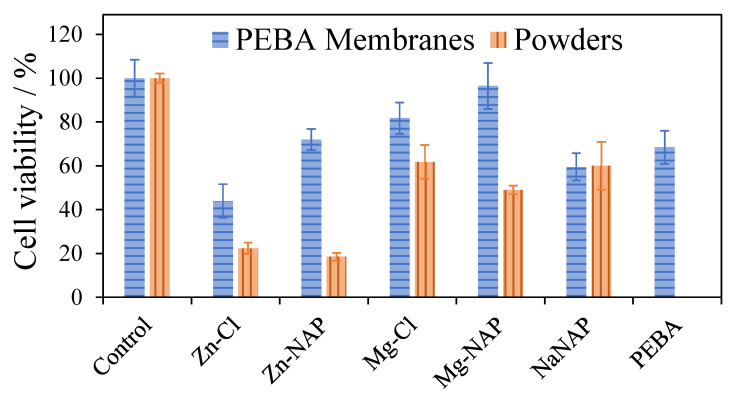
MTT test for NHDF cells in contact with pristine PEBA and PEBA composite membranes containing Zn-Cl, Zn-NAP, Mg-Cl, Mg-NAP or NaNAP particles (in blue–horizontal lines) and powdered samples of Zn-Cl, Zn-NAP, Mg-Cl, Mg-NAP and NaNAP (in orange–vertical lines) for 3 h. Error bars represent standard error (n = 6).

**Figure 8:**
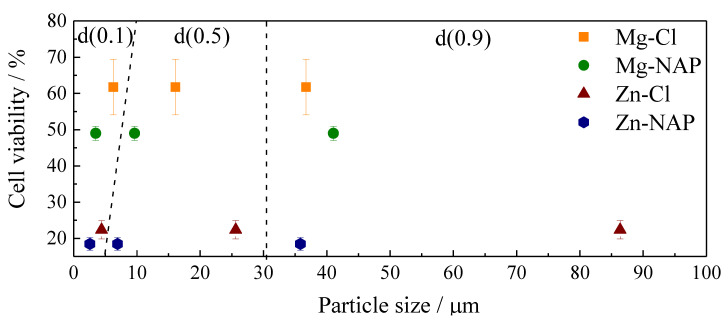
Cell viability percentage of LDH powder samples according to particle size expressed by d(0.1), d(0.5) and d(0.9) values (from Figure S3), related to the percentage of particles that present up to the corresponding sizes.

Cell viability percentages for PEBA membranes and powdered samples on NHDF cells are shown in Figure 7. LDHs and their composites containing Mg^2+^ presented higher cellular viability percentages compared to the materials composed by Zn^2+^, whose tendency is supported by previous works [54,55]. For powdered LDHs, cell viability was also higher when intercalated with Cl^−^ anions (mainly for magnesium LDH). The relation among LDH particle sizes and composition with cell viability can be seen in Figure 8. All LDH-PEBA membranes presented higher cellular viability compared to the respective powdered LDHs samples. The viability of PEBA_NaNAP membrane and powder NaNAP are comparable. PEBA_Zn-NAP, PEBA_Mg-Cl and PEBA_Mg-NAP display an improved biological response in comparison to pristine PEBA, even more pronounced for hybrid LDHs, which can be related to the favored particle dispersion into PEBA when NAP is intercalated, as earlier discussed. Once more, the PEBA_Mg-NAP sample stands out, exhibiting the greater viability percentage among all samples (96%). The NAP intercalated into the Mg-LDH, dispersed in the polymeric, decreases the cytotoxicity of the drug in comparison with NaNAP powder and the PEBA_NaNAP composite. Moreover, a synergistic effect was observed, since the cell viability of PEBA_Mg-NAP was higher than the ones observed for pristine PEBA, NaNAP salt or Mg-NAP LDH. 

## 4. Discussion

Hybrid organic–inorganic LDHs experienced an improved interaction with PEBA. Such improvement was reflected on membrane thicknesses, lower for the PEBA_Mg-NAP and PEBA_Zn-NAP compared to the Mg-Cl and Zn-Cl samples. The gain in particle–polymer chain interaction compared to the Cl-LDHs is in consonance with the superficial morphology analyzed by SEM. Although surface fill failures are observed for the PEBA_Zn-NAP sample, LDH particle aggregation is not visualized, which is different to the PEBA_Mg-Cl and PEBA_Zn-Cl membranes. As normally observed [11,43], Zn^2+^ cations conferred a higher structural organization to the LDH structure, which possibly contributed to the visualization of the characteristic plate-like morphology of Zn-Cl particles in the SEM image of the PEBA_Zn-Cl membrane. The smoothness of the PEBA_Mg-NAP sample is compared to that of the pristine PEBA. Lower average particle sizes, verified for NAP-LDHs, resulted in a better particle distribution. However, the lower polydispersity of the Mg-LDH helps to explain its unpaired performance. Though Zn-NAP LDH presents a higher structural organization than the Mg-NAP LDH, verified by XRD, the absence of peaks related to LDH phase could also indicate a more effective dispersion of particles in the PEBA_Mg-NAP membrane, which is consonant with its improved homogeneity and surface smoothness verified by SEM. The superior Mg-NAP interaction with PEBA is once again reinforced by the greater mechanical performance of the PEBA_Mg-NAP membrane. This inference is also supported by the opposite behavior of the PEBA_Zn-Cl sample that experienced inferior mechanical performance and showed an elevated Zn-Cl particle aggregation, verified by optical microscopy and SEM, and suggested by XRD data. Independently of the homogeneity of particle distribution into PEBA or the particle nature, LDH or NaNAP salt, the very close T_g_ values show that the particles did not cause a disorder in the polymer chains, frequently observed for polymer composites by the decrease in T_g_ values in relation to pristine polymer [28]. 

Concerning the in vitro NAP release profiles, the NAP release rate can be modulated by changing the nature of the divalent cation in the LDH composition. Retarded NAP release from Zn_4_FeAl-NAP LDH in comparison to the Mg_4_FeAl-NAP, same compositions applied in this work, was also observed in our previous work [11], whose in vitro NAP release was performed from tablets and the assay conducted in a dissolution tester in phosphate buffer solution (pH equal to 7.4). In such work, after 10 h, 30 and 59% of NAP release were observed for Zn-LDH and Mg-LDH, respectively. Differently from the results shown herein, NaNAP dissolution lasted for only 30 min. Therefore, PEBA can be a good support for NaNAP particles to sustain drug release. As discussed earlier, PEBA_Mg-NAP sample characterization indicates a more homogeneous distribution of particles in the polymeric matrix. Therefore, related to the other samples, an improvement in the contact area of the particles with the release medium is expected, which can explain its higher k value. On the other hand, higher Zn-NAP and NaNAP particle aggregation leads to small k values. As observed in another work [11,43], the lower amount of released M^3+^ (Fe^3+^ or Al^+3^) cations is out of the limit of detection from the same quantitative method. The higher solubility of Mg^2+^ in comparison with Zn^2+^ is directly related to the solubility product (Kps) values of the isolated hydroxides, as follows: Mg(OH)_2_ > Zn(OH)_2_ > Al(OH)_3_ > Fe(OH)_3_ [56].

For both powdered LDH and LDH membranes, cell viability was higher in the presence of LDH intercalated with Cl^−^ anions. Despite the nature of the intercalated anion, in the case of suspensions, toxicity has shown a dependence on surface area. Small particle sizes with high superficial areas tend to overload cells [54]. In fact, LDHs intercalated with NAP presented lower average particle sizes and lower cell viabilities. Several papers report the in vitro cytotoxicity of LDHs by MTT method. Some of these papers are briefly discussed hereafter. Kura et al. [57] analyzed the cytotoxicity of the Zn/Al-NO_3_ LDH and the same layer composition intercalated with levodopa, an anti-Parkinsonian compound. Concentrations of levodopa, pristine and hybrid-LDH equal to 5, 10 50, 100 and 150 µg mL^−1^ were applied to mouse embryonic fibroblasts (3T3 cells). Cell viability decreased progressively with the increase in levodopa and LDH materials concentration. Although the hybrid LDH has reached the highest cell viability percentage for the higher tested concentration compared to non-loaded levodopa and the pristine LDH, the value was reduced for about 40% compared to the lower applied concentration. Saifullah et al. [58] studied the in vitro cytotoxicity of the Mg/Al-NO_3_ LDH and the corresponding LDH intercalated with isoniazid, a drug used for the treatment of tuberculosis, in contact with normal human lung fibroblast and 3T3 cells. By increasing material concentration from 0.781 to 50 µg mL^−1^, a reduction of about 20% in the cell viability of both materials is noted. Mohsin et al. [59] evaluated the cytotoxicity of the Zn/Al LDH intercalated with NO_3_^−^ anions and with a mixture of two ultraviolet-ray absorbers benzophenone 4 and Eusolex^®^ 232, applying dermal fibroblast cells. Pristine and hybrid LDHs do not present significant toxicity up to 25 µg mL^−1^, however, cell viabilities decreased for about 50% by doubling materials concentration. Pagano et al. [55] studied the intercalation of folate into Zn/Al and Mg/Al LDHs, aiming topical applications for treatment of aged and photo-damaged skin. Cytotoxicity essays were carried on human keratinocytes and human primary dermal fibroblasts. Zn-LDH was more toxic than Mg-LDH, as observed in this work. For the Mg-LDH, the cell viability percentage varied from about 60% to 30% by increasing material concentration (related to the content of loaded folate) from 215 to 1529 µg mL^−1^. Moreover, at the tested condition, intercalation into LDH did not show an advantage concerning the cytotoxicity once cells treated with folic acid presented cell viability of about 80% or more for all concentrations and, once again, such results are in agreement with the ones presented here. The NAP concentration applied in this work for verification of the in vitro cytotoxicity when present in the NaNAP salt, loaded on LDHs or in the respective PEBA composites is around 1000 µg mL^−1^ and cell viability was below 70% for samples in powder. The statement of the cytotoxicity depends on the material’s concentration and, as discussed above, the biocompatibility of LDHs is restricted to concentrations in the order of tenths or hundredths µg mL^−1^. As shown in this work, this limitation can be overcome by embedding LDH particles in a polymeric matrix, reducing the dependence of particle size and concentration in the material’s cytotoxicity.

The PEBA_Mg-NAP membrane presented a sufficient or greater performance in all aspects evaluated in this work. As proposed, a multifunctional membrane was designed based on a polymeric support whose mechanical performance was improved, containing LDH particles aiming for adequate LDH compositions for biological application (containing a larger content of essential metals in the compositions). The engendered material was able to sustain the release of a bioactive organic species and also Mg^2+^ cations as well as a great in vitro biocompatibility. We believe that this work may advance the application of iron-based LDH and PEBA composites in general devices to support tissue regeneration. 

## 5. Conclusions

The intercalation of NAP into LDH improved the polymer–LDH interaction, enhancing considerably the mechanical performance of PEBA. Drug release could be modulated by changing the chemical composition of the membranes; NAP release was sustained for at least 8 h by all formulations. Developed PEBA_Mg-NAP composite was considered the ideal formulation, among all membranes developed in this work, to act as therapeutic dressing; once it was able to sustain drug release, it showed greater mechanical resistance and improved cell viability if compared to the performance of separated components (PEBA, NAP and LDH).

However, in vitro and in vivo studies are necessary to evaluate the influence at cellular level of PEBA_Mg-NAP in contact with wounds and in the wound healing process. Additionally, this study encourages us to explore the intercalation of other bioactive species into LDH aimed at making improvements in wound healing process.

## Figures and Tables

**Table 1:** Weight percentages of LDH and NAP in the membranes.

Components of the Membranes	LDH wt%	NAP wt%
NaNAP	-----	4
Mg-Cl	10	-----
Mg-NAP	10	4
Zn-Cl	13	-----
Zn-NAP	13	4

**Table 2:** Thickness, water static contact angle, stress and strain at break and Young modulus of pristine PEBA membrane and PEBA composites.

Sample	Thickness (µm)	Contact Angle (°)	Stress at Break (kPa)	Strain at Break (%)	Young Modulus (MPa)
PEBA	82 ± 14	78 ± 1	479 ± 27 ^(a)^	22 ± 7 ^(a)^	3.5 ± 0.9
PEBA_NaNAP	90 ± 10	60 ± 5	550 ± 14 ^(a)^	15 ± 7 ^(a)^	7.4 ± 0.8
PEBA_Mg-Cl	100 ± 1	73 ± 1	535 ± 49	35 ± 9	3.9 ± 0.5
PEBA_Mg-NAP	86 ± 6	74 ± 1	829 ± 45	55 ± 16	5.3 ± 0.7
PEBA_Zn-Cl	103 ± 5	75 ± 2	379 ± 120 ^(a)^	22 ± 8 ^(a)^	3.4 ± 0.4
PEBA_Zn-NAP	96 ± 6	75 ± 1	632 ± 129 ^(a)^	26 ± 2 ^(a)^	5.2 ± 0.7

^(a)^ ref. [38].

**Table 3:** IR wavenumbers (in cm^−1^) indicated in the spectra of PEBA membrane and PEBA composites and tentative assignments.

Wavenumber	Assignment	Wavenumber	Assignment ^1^
3440	ν O–H	1393	ν C–C (naphthalene), ω CH_3_
3300	ν N–H	1368	δ C–C–H
2920	ν_a_ CH_2_	1360	ν_3_ CO_3_^2−^
2850	ν_s_ CH_2_	1242	ν C–N–H
1735	ν C=O (O–C=O)	1162	ν C–O
1638	ν C=O (H–N–C=O)	1104	ν_a_ C–O–C
1631	ν C–C=C	925	γ =C–H
1541	ν C–N	815	γ =C–H
1467	δ CH_2_	721	δ CH_2_ group (–(CH_2_)_n_–, n > 4)
1447	sc CH_2_	<700	Mg-OH or Zn-OH translations

^1^ ν = stretching, ν_a_ = antisymmetric stretching, ν_s_ = symmetric stretching, δ = bending, β = bending in plane, γ = bending out of plane, sc = scissoring and ω = wagging.

**Table 4:** Release velocity constant (k), exponent of release (n) and coefficient of determination (*R*^2^) obtained from the application of Power Law kinetic release model.

Sample	k (h^−n^)	n	*R* ^2^
PEBA_Mg-NAP	0.216	0.375	0.945
PEBA_NaNAP	0.053	0.762	0.985
PEBA_Zn-NAP	0.040	0.626	0.969

## Supplementary Materials

The following are available online at https://www.mdpi.com/1999-4923/12/11/1130/s1, Table S1: Elemental chemical composition of M_4_FeAl-Cl and M_4_FeAl-NAP LDHs (M = Mg^2+^ or Zn^2+^), Table S2: Peak glass transition (Tg PE), crystallization (TC PE) and melting (Tm PE) temperatures obtained from DSC analyses of pristine PEBA and PEBA composites, Figure S1: Representative pictures of water static drop deposited on the surface of PEBA and PEBA composite membranes, Figure S2: Apparent volume occupied by the same mass of each LDH, Figure S3: LDHs Particles size distribution in 2-propanol, Figure S4: FT-IR spectra of pristine PEBA and PEBA composites containing Cl-LDHs (a) and composites containing the NaNAP salt and hybrid organic–inorganic LDHs (b), Figure S5: TGA and MS curves of pristine PEBA membrane, Figure S6: TGA and MS curves of PEBA reagent (beads).
