# Supplementary material for: Polymer/Iron-Based Layered Double Hydroxides as Multifunctional Wound Dressings
Source: Pharmaceutics. 2020 Nov 23;12(11):1130. doi: 10.3390/pharmaceutics12111130 (PMC7700130; doi:10.3390/pharmaceutics12111130)
Supplement: Supplementary file 1 [file pharmaceutics-12-01130-s001.pdf]

pharmaceutics

Supplementary Materials: Polymer/Iron-Based Layered Double Hydroxides as Multifunctional Wound Dressings

Mariana Pires Figueiredo, Ana Borrego-Sánchez, Fátima García-Villén, Dalila Miele, Silvia Rossi, Giuseppina Sandri, César Viseras and Vera Regina Leopoldo Constantino

Table S1. Elemental chemical composition of M_4FeAl-Cl and M_4FeAl-NAP LDHs (M = Mg^{2+} or Zn^{2+}).

Sample	M/Al	M/Fe	Fe/Al	M / (Fe + Al)	Wt % C	Wt % NAP
Mg_4FeAl-Cl	4.32	4.09	1.06	2.10	----	----
Mg_4FeAl-NAP	4.29	4.10	1.05	2.10	26.64	36.35
Zn_4FeAl-Cl	3.53	3.47	1.02	1.75	----	----
Zn_4FeAl-NAP	3.64	3.53	1.03	1.79	19.41	26.48

Table S2. Peak glass transition ($T_{g\,PE}$), crystallization ($T_{C\,PE}$) and melting ($T_{m\,PE}$) temperatures obtained from DSC analyses of pristine PEBA and PEBA composites.[1.]

Material	$T_{g\,PE}$ (°C)	$T_{c\,PE}$ (°C)	$T_{m\,PE}$ (°C)
PEBA	-60	-14	21
PEBA_NaNAP	-59	-15	20
PEBA_Zn-Cl	-59	-15	20
PEBA_Zn-NAP	-59	-16	20
PEBA_Mg-Cl	-59	-16	21
PEBA_Mg-NAP	-60	-16	19

[1]$T_{g\,PE}$ – glass transition temperature of PE portion, $T_{c\,PE}$ – crystallization temperature of PE portion, $T_{m\,PE}$– melting temperature of PE portion.

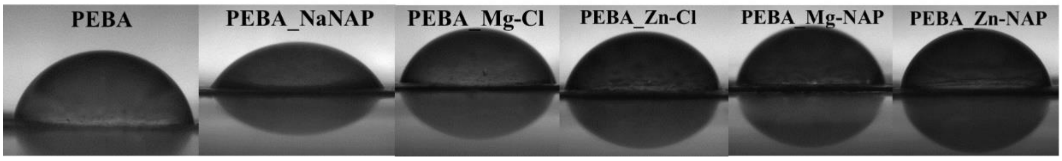

Figure S1. Representative pictures of water static drop deposited on the surface of PEBA and PEBA composite membranes.

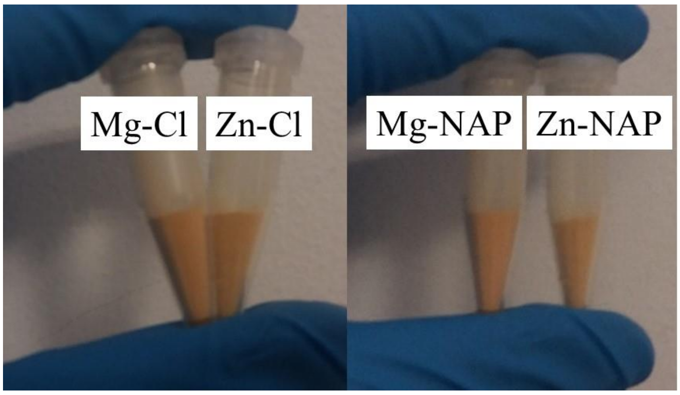

Figure S2. Apparent volume occupied by the same mass of each LDH.

Mg-Cl

d(0.1)= 6.273 μm d(0.5)= 16.093 μm d(0.9)= 36.719 μm

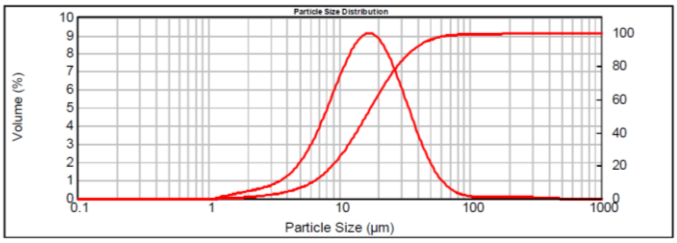

Mg-NAP

d(0.1)= 3.495 μm d(0.5)= 9.631 μm d(0.9)= 41.039 μm

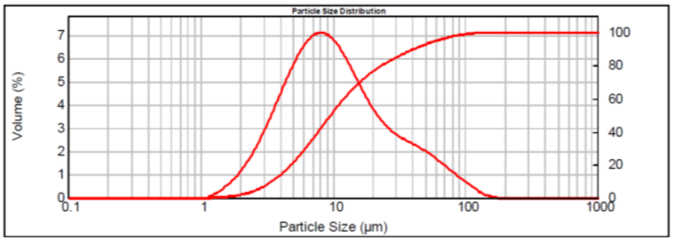

Zn-Cl

d(0.1)= 4.414 μm d(0.5)= 25.617 μm d(0.9)= 86.357 μm

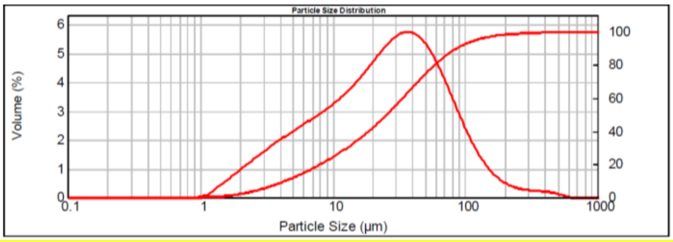

Zn-NAP

d(0.1)= 2.566 μm d(0.5)= 6.928 μm d(0.9)= 35.830 μm

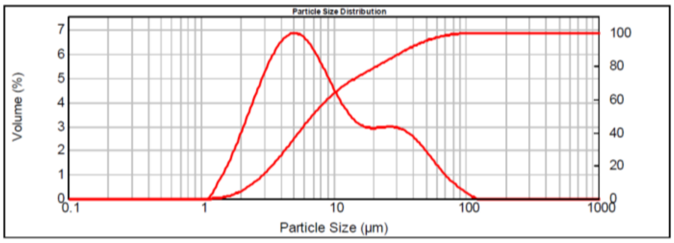

Figure S3. LDHs Particles size distribution in 2-propanol.

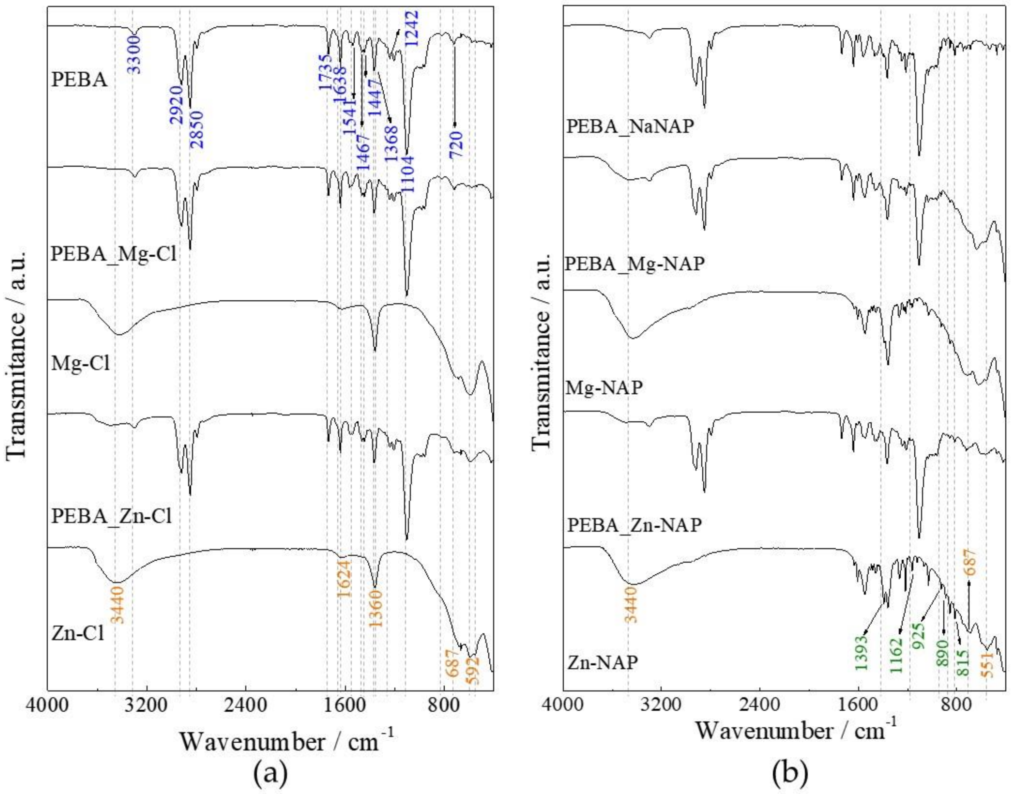

Figure S4. FT-IR spectra of pristine PEBA and PEBA composites containing Cl-LDHs (a) and composites containing the NaNAP salt and hybrid organic-inorganic LDHs (b).

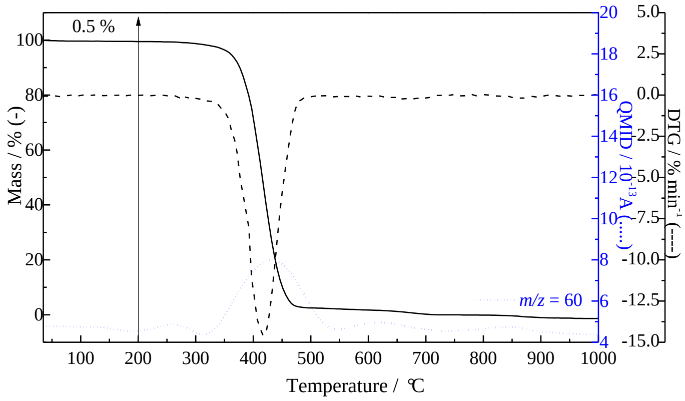

Figure S5. TGA and MS curves of pristine PEBA membrane.

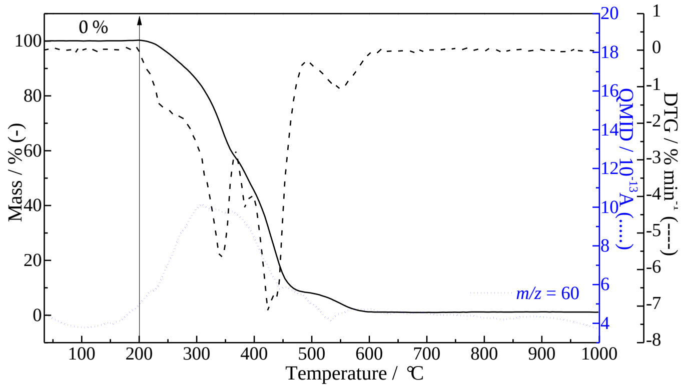

Figure S6. TGA and MS curves of PEBA reagent (beads).
